# The role of TNF-α in the fate regulation and functional reprogramming of mesenchymal stem cells in an inflammatory microenvironment

**DOI:** 10.3389/fimmu.2023.1074863

**Published:** 2023-02-06

**Authors:** Weiqiang Li, Qianqian Liu, Jinchao Shi, Xiang Xu, Jinyi Xu

**Affiliations:** ^1^ Department of Stem Cell & Regenerative Medicine, State Key Laboratory of Trauma, Burn and Combined Injury, Daping Hospital, Army Medical University, Chongqing, China; ^2^ Department of Research and Development, Ankerui (Shanxi) Biological Cell Co., Ltd., Shanxi, China; ^3^ Department of Biochemistry and Molecular Biology, College of Basic Medical Science, Army Medical University, Chongqing, China; ^4^ State Key Laboratory of Ophthalmology, Zhongshan Ophthalmic Center, Sun Yat-sen University, Guangzhou, China

**Keywords:** mesenchymal stem cells, TNF-α, exosomal microvesicles, immunomodulation, tissue regeneration and repair

## Abstract

Mesenchymal stem cells (MSCs) are pluripotent stem cells with multidirectional differentiation potential and strong immunomodulatory capacity. MSCs have been widely used in the treatment of injured, inflammatory, and immune-related diseases. Resting MSCs lack differentiation and immunomodulatory ability. Instead, they rely on microenvironmental factors to: 1) stimulate and regulate their expression of specific cell growth factors, chemokines, immunomodulatory factors, or receptors; or 2) direct their differentiation into specific tissue cells, which ultimately perform tissue regeneration and repair and immunomodulatory functions. Tumor necrosis factor (TNF)-α is central to the creation of an inflammatory microenvironment. TNF-α regulates the fate and functional reprogramming of MSCs, either alone or in combination with a variety of other inflammatory factors. TNF-α can exert opposing effects on MSCs, from inducing MSC apoptosis to enhancing their anti-tumor capacity. In addition, the immunomodulation and osteogenic differentiation capacities of MSCs, as well as their exosome or microvesicle components vary significantly with TNF-α stimulating concentration, time of administration, or its use in combination with or without other factors. Therefore, this review discusses the impact of TNF-α on the fate and functional reprogramming of MSCs in the inflammatory microenvironment, to provide new directions for improving the immunomodulatory and tissue repair functions of MSCs and enhance their therapeutic potential.

## Introduction

Mesenchymal stem cells (MSCs) are important members of the stem cell family and are found in a variety of tissues of mesodermal origin, such as the bone marrow, umbilical cord, placenta, amniotic membrane, dental pulp, and adipose tissue (1). MSCs can directionally differentiate into lineages of tissue cells, including chondrocytes, adipocytes, and osteoblasts et al. On doing so, MSCs indirectly facilitate the regeneration and repair of damaged tissues by tissue replacement and cell growth factor secretion. Moreover, MSCs also directly influence the proliferation, activation, and polarization of T cells, B cells, dendritic cells (DCs), and natural killer (NK) cells by secreting immunomodulatory factors or expressing membrane receptors, resulting in their immunosuppression or immunoregulation. However, resting MSCs have no differentiation or immunomodulatory capacity, and only gain these functions after they are stimulated by microenvironmental modulators, particularly immune or inflammatory factors (2).

Targeted activated lymphocytes induce the apoptosis of MSCs by secreting tumor necrosis factor (TNF)-α, one of the main immune factors regulating the fate and function of MSCs (3). TNF-α belongs to the TNF superfamily, which accounts for approximately 70%–95% of the biological activity of TNFs, and is mostly produced by activated macrophages and immune cells such as lymphocytes (4). TNF-α plays an important role in inflammation, immune response regulation, and carcinogenesis. It is a key signaling molecule, which coordinates immune and inflammatory responses and can initiate cell apoptosis and programmed necrosis. Recently, researchers found that while a highly inflammatory environment inhibits the function of MSCs, modulation of MSCs by certain inflammatory factors might play a positive role in the treatment of many diseases (5). TNF-α is the main inflammatory factor in the microenvironment of inflammatory disease. Therefore, the question of whether TNF-α can indirectly influence the disease process or the clinical therapeutic efficacy of MSCs by regulating tissue regeneration and repair or the immunoregulatory function of MSCs needs to be addressed. Numerous studies have reported either positive or negative regulatory effects of TNF-α on the fate and function of MSCs. Dorronsoro et al. found that inhibition of the NF-κB signaling pathway by silencing IκB kinase β (IKK-β) and TNF-α receptors could significantly weaken the immunosuppressive capacity of MSCs (6). However, López-García and colleagues claimed that inflammatory factors such as TNF-α and interferon (IFN)-γ positively or negatively affect the immunomodulatory function of MSCs by modulating specific immunoregulatory molecules in their exosomes, microvesicles or micro(mi)-RNAs (7). Here, we summarize and discuss the latest research exploring the different regulatory mechanisms linking TNF-α and MSCs. We envisage that this review will offer new insights to help improve the clinical efficacy of TNF-α-modulated MSCs for the treatment of various inflammatory or immune-related diseases.

## Methods

### Search engines and time period

An electronic search concerning the role of TNF-α in the regulation and functional reprogramming of MSCs in an inflammatory microenvironment was conducted in NCBI (PubMed) and Web of Science. Articles with full publications that appeared in English language biomedical journals and published between 1 January 1995 and 18 December 2022 were included in the current review.

### Search strategy

**Figure 1:**
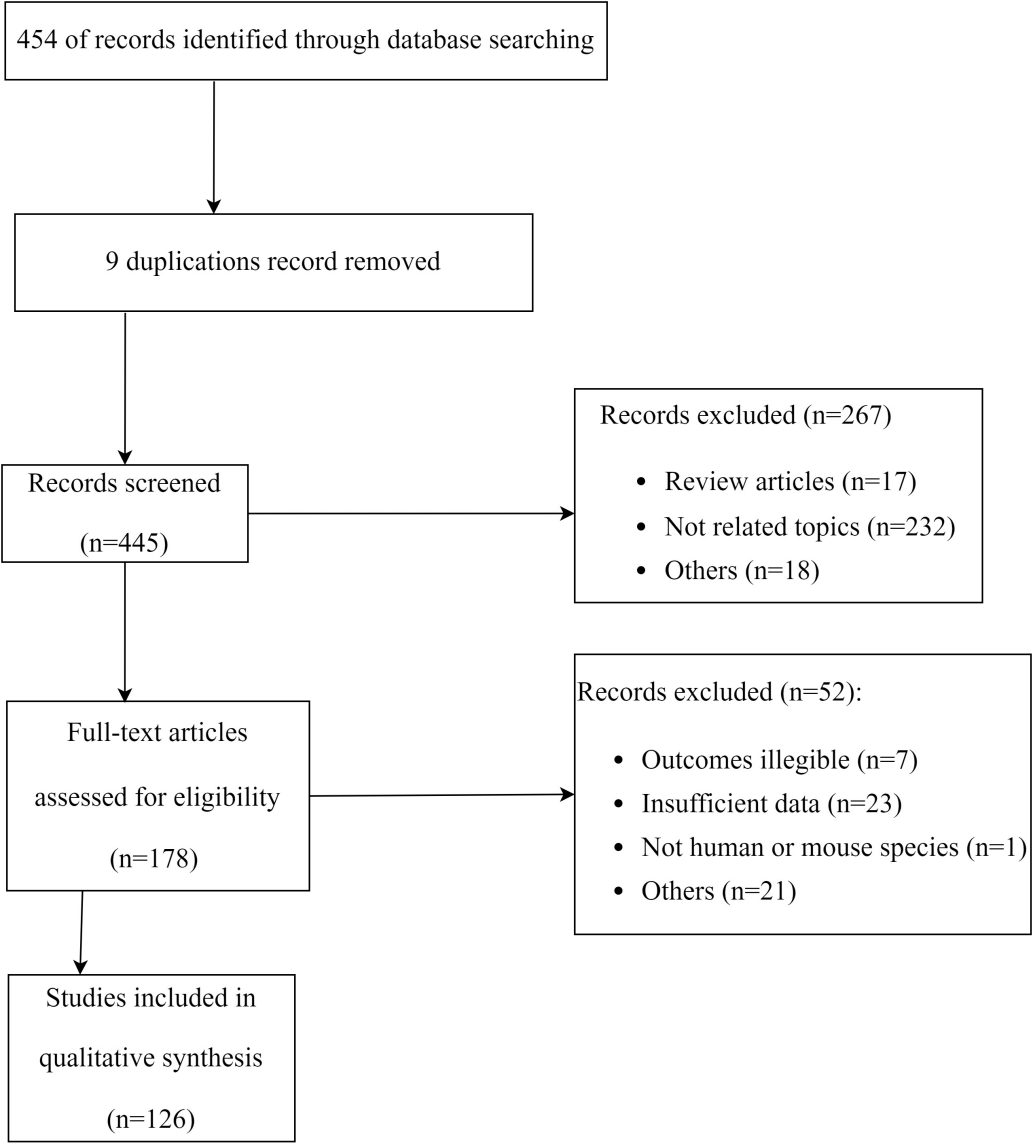
Flowchart of the systematic search and screening.

Public databases were searched using the combination of keywords ‘TNF-α‘ and ‘mesenchymal stem cells’ or ‘MSCs’in the Titles/Abstract of publications. This provided the initial database for our review. Initial search was performed in late June 2022, followed by two searches in September 2022, and a final update in December 2022. The PRISMA flow diagram for the literature search process was illustrated in **Figure 1**.

### Selection criteria

Eligibility criteria for inclusion were: a) prospective clinical studies; b) animal studies; c) cell studies; d) studies reporting effect of TNF-α on MSCs in an inflammatory microenvironment; e) studies in the English language. Review articles (narrative and systematic), short communications, letters to editors, and data extraction were excluded. All publications were screened according to the PRISMA guideline. PRISMA 2020 checklist was used to assess the quality of our review.

### Data synthesis

Statistics derived from each paper were summarized by pictures and tables involving the concentration of inflammatory cytokines, species, and type of MSCs and action of exchanged exosomes or microvesicles in tissue.

## TNF-α inhibits MSC proliferation, while promoting their autophagy and apoptosis

The inflammatory microenvironment is a complex system, involving: 1) pro-inflammatory cells and factors such as M1 macrophages, CD4^+^ T cells, CD8^+^ T cells, interleukin (IL)-1β, IL-2, IL-6, IL-8, IL-12, IL-17, TNF-α, and IFN-γ; and 2) anti-inflammatory cells and factors such as M2 macrophages, IL-4, IL-10, IL-35, and tumor growth factor (TGF)-β (8, 9). *In vitro* experiments often simulate the occurrence, development, and inhibition of the inflammatory environment *in vivo* using combinations of pro-inflammatory factors, especially TNF-α and IFN-γ.

### TNF-α inhibits MSC proliferation in an inflammatory environment

As an initiating factor of the inflammatory response, TNF-α plays a prominent role in the fate regulation and functional reprogramming of MSCs. In the inflammatory microenvironment, TNF-α acts in concert with many other inflammatory factors. Domenis et al. treated human adipose MSCs with 10–40 ng/ml TNF-α and IFN-γ for 48 h and found that the morphology and proliferative capacity of MSCs was altered and reduced, respectively, with the increase in cytokine dose (9). Similarly, Crop et al. demonstrated that after human adipose MSCs were stimulated for 7 days with a pro-inflammatory mixture of 20 ng/ml TNF-α, 50 ng/ml IFN-γ, and 10 ng/ml IL-6, their diameter increased, while their proliferative capacity declined (10). This indicates that treating hMSCs with TNF-α and IFN-γ affects their morphology and proliferation ability.

### TNF-α induces MSC autophagy

**Figure 2:**
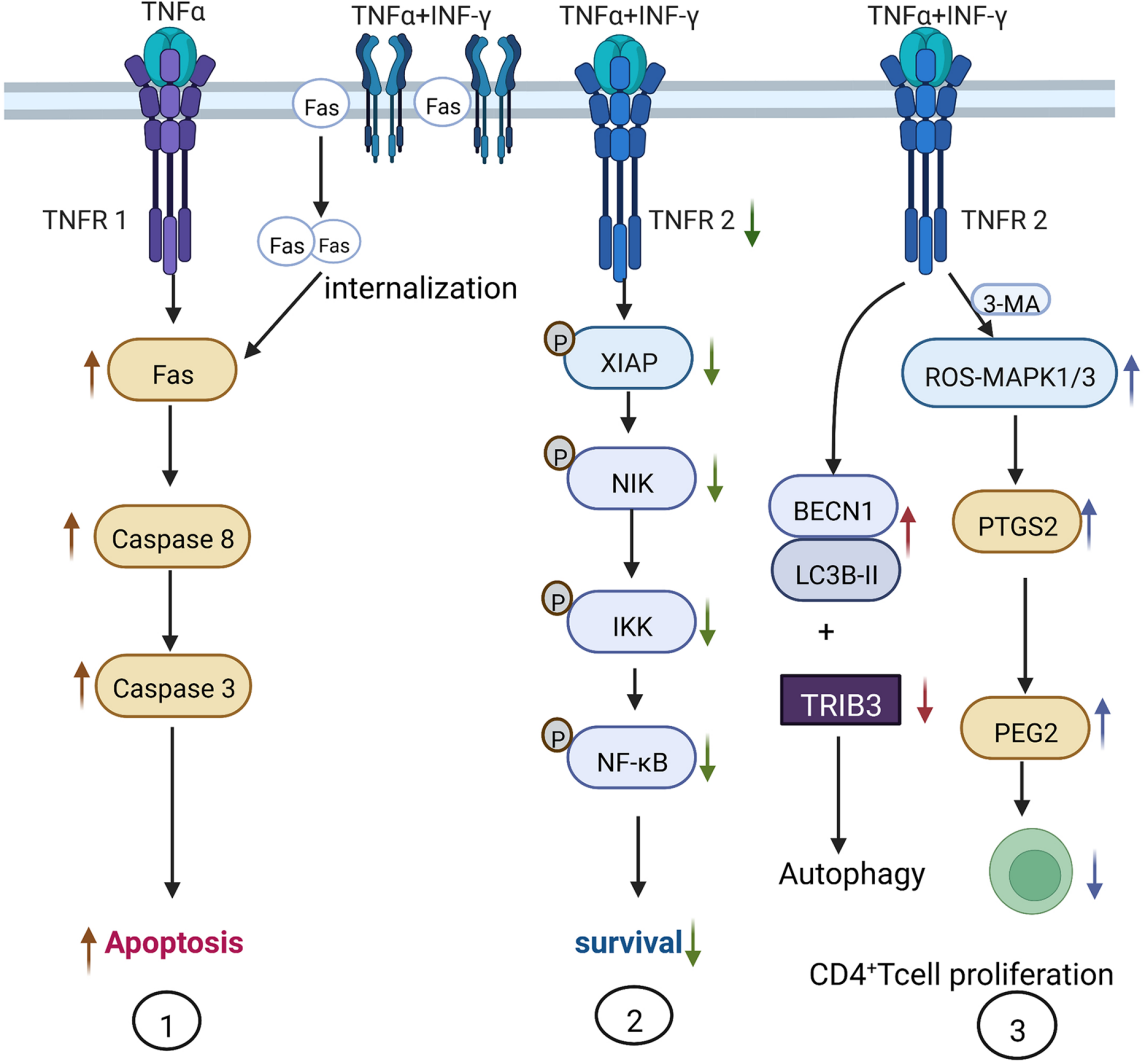
Mechanism of TNF-α-induced autophagy and apoptosis in MSCs. 1) IFN-γ assists TNF-α in the induction of apoptosis *via* TNFR1. 20–200 ng/ml of TNF-α induces MSC apoptosis in a dose-dependent manner, while 50 ng/ml IFN-γ only promotes the expression of Fas on the cell surface. Adding 20 ng/ml TNF-α promotes Fas internalization into cells to induce MSC apoptosis. 2) TNF-α and IFN-γ inhibit TNFR2 and the downstream anti-apoptotic signaling pathway. When MSCs are treated with 20 ng/ml TNF-α and 50 ng/ml IFN-γ, the phosphorylation of XIAP and NF-κB within MSCs is inhibited and the anti-apoptotic effect is suppressed. 3) Administration of 20 ng/ml TNF-α and 50 ng/ml IFN-γ increases the expression of autophagy-related genes *BECN1* and *LC3B-II*, and decreases the expression of autophagy-inhibiting molecule TRIB3, thus increasing autophagy; 3-methyladenine (3-MA) can inhibit autophagy by activating the ROS-MAPK1/3 pathway and inhibiting CD4^+^ T cell proliferation by stimulating MSCs to secrete PGE-2.

Autophagy is a process that occurs under conditions of starvation and growth factor deficiency, in which cells survive by degrading their own components or organelles (11). *ATG5 (* 12), *ATG7*, *LC3 (* 13), and *BECN1* are important autophagy-associated genes, which are expressed in fibroblasts, lymphocytes, and MSCs. Dang et al. found that stimulating bone marrow mice MSCs for 4 h with 20 ng/ml TNF-α significantly increase their mRNA and protein expression of *BECN1*. Meanwhile, stimulation of the MSCs with 50 ng/ml IFN-γ only resulted in the upregulation of Becn1 mRNA and not protein expression. However, treating the MSCs with a combination of 20 ng/ml TNF-α and 50 ng/ml IFN-γ produced the highest rise in *BECN1* mRNA and protein expression, in comparison with each treatment alone (14) (**Figure 2**). These experiments demonstrate that TNF-α is a key factor in the induction of autophagy in bone marrow MSCs, while IFN-γ plays a synergistic role.

Similarly, 20 ng/ml TNF-α stimulation of human umbilical cord MSCs for 24 h increased the expression of autophagy-related protein microtubule-associated protein 1 light chain B-II (LC3B-II) and lowered the expression of autophagy inhibitory molecule tribbles homology protein 3 (TRIB3) (15). Therefore, in the inflammatory microenvironment, and especially during *in vitro* experiments, the presence of high concentrations of inflammatory factors promotes the survival of MSCs *via* autophagy.

### TNF-α induces MSC apoptosis

TNF-α is mainly produced by macrophages and participates in various pathophysiological activities such as inflammatory cytokine production, cell survival, proliferation, and apoptosis. TNF-α performs these functions on binding to its receptors: tumor necrosis factor receptor 1 (TNFR1) and tumor necrosis factor receptor 2 (TNFR2) on the cell membrane. Li et al. treated human umbilical cord MSCs with 20 ng/ml TNF-α and 50 ng/ml IFN-γ and reported a significant upregulation in the expression of MCP and IL-6, which eventually inhibited the protein synthesis of the inhibitor of apoptosis (IAP), leading to the mass apoptosis of MSCs (16). Furthermore, Liu and colleagues found that 0–200 ng/ml IFN-γ did not induce mice MSC apoptosis, while the combination of 20 ng/ml TNF-α and 50 ng/ml IFN-γ induced MSC apoptosis more effectively than using 20 ng/ml TNF-α alone. Therefore, IFN-γ enhances the pro-apoptotic action of TNF-α. The synergistic mechanism may involve IFN-γ upregulating the expression of Fas on the cell membrane. TNF-α could then promote Fas internalization, thus enhancing Caspase-3- and Caspase-8-mediated apoptotic signaling. Alternatively, IFN-γ also decreases TNFR2 expression and the phosphorylation of NF-κB, the X-linked inhibitor of apoptosis protein (XIAP), κB kinas (IKK), and FLICE inhibitory protein (FLIP), which inhibits the anti-apoptotic pathway of MSCs and eventually results in apoptosis (17) (**Figure 2**).

Since the high concentration of TNF-α in the inflammatory environment induces MSC autophagy and apoptosis and affects MSC regeneration, immune regulation, and differentiation functions, the non-specific autophagy inhibitor 3-methyladenine (3-MA) has been used in animal experimental models to inhibit MSC autophagy. 3-MA inhibits MSC autophagy *via* the activation of the ROS-MAPK1/3 pathway, significantly reducing the mRNA and protein expression of TNF-α, IFN-γ, IL-6, and IL-17. At the same time, 3-MA also upregulates the production of a key immune regulatory factor prostaglandin E2 (PGE-2) (downstream from PTGS2), thereby restoring the immunomodulatory function of MSCs to suppress CD4^+^ T cell proliferation in nude mice (14).

Similarly, aspirin and etanercept can reduce the impact of the inflammatory environment on the apoptosis and proliferation of bone marrow MSCs by inhibiting the functions of TNF-α and IFN-γ. These drugs can restore the self-renewal and multidirectional differentiation capacity of MSCs, and have been shown to be effective in treating acute graft versus host disease and cranial defects in animal experiment (18, 19). Furthermore, Zhao et al. used a lentiviral vector (GV358) to overexpress TNFRII-Fc in human umbilical cord MSCs, neutralizing the effects of TNF-α on the apoptosis and autophagy of these cells (16).

The inflammatory environment is an important regulator of MSC function. Nevertheless, prolonged chronic inflammation or the high concentration of TNF-α inhibit the proliferation of MSCs, while promoting their autophagy or apoptosis. Therefore, in the next section we evaluate the effect of different TNF-α concentrations and administration time points on the proliferation, autophagy, and apoptosis of MSCs and provide guidelines for future clinical therapies.

## TNF-α enhances the immunomodulatory capacity of MSCs

TNF-α is mainly expressed by DCs, macrophages, NK cells, and T cells, and is mostly secreted in the inflammatory environment, participating in processes such as tissue regeneration and immunomodulation (20). In the development of inflammation, TNF-α is the first factor secreted by immune cells, which can enhance or weaken the effects of other cytokines (21, 22). In the inflammatory microenvironment, the immunomodulatory capacity of MSCs is susceptible to various factors, which leads to them either promoting or reducing inflammation. MSCs exert their immunomodulatory effects mainly through direct cellular contact or the secretion of growth factors, cytokines, and chemokines.

Resting mouse MSCs constitutively express low levels of COX-2, PGE-2, TGF-β1, HGF (23) and B7H1 (24), meanwhile resting human MSCs can persistently express ICAM-1 (25), CXCL10 (26), IL-6 (26), IL-8 (27), CCL-2 (28), PD-L1 (29), MHC I (30) and IDO (31). However, in both human and mouse, either TNF-α or IFN-γ alone can increase the protein production of COX-2 and PGE-2, while PD-L1 (29, 32) and IDO (31) can only be induced by IFN-γ alone (33)in inflammatory environment.

Several articles have shown that TNF-α can enhance the immunomodulatory capacity of MSCs or to assist other cytokines to play immunomodulatory roles in the inflammatory environment. Because of the large variety of immune cells and cytokines, and the complex interplay between them, in this review, we will focus on summarizing the mechanisms by which TNF-α modulates MSC function.

### TNF-α stimulation alone enhances the immunosuppressive capacity of MSCs

#### TNF-α stimulates MSCs to suppress the inflammatory response by promoting polarization of anti-inflammatory M2 macrophages

Macrophages are subdivided into pro-inflammatory M1 macrophages and anti-inflammatory M2 macrophages. The M1 subtype eliminates nonself-components, prevents tumor growth, and the mediates T helper (Th)1-mediated pro-inflammatory response by releasing pro-inflammatory factors such as IL-1β, TNF-α, IL-12, IL-6, and IL-23. The M2 subset is involved in Th2-mediated immune regulation by secreting anti-inflammatory factors such as IL-10 (8).

It has been reported that the coculture of human bone marrow MSCs pre-stimulated with 10 ng/ml TNF-α and macrophages promoted the release of IL-10 from M2 macrophages and reduced TNF-α levels in the culture medium by increasing the secretion of COX-2 and PGE-2 (33, 34).

#### TNF-α enhances the immunosuppressive capacity of MSCs by inducing TSG-6 expression

Tumor necrosis factor alpha stimulated gene-6 (TSG-6) is a key anti-inflammatory molecule synthesized by MSCs following the high expression of the *TNFA1P6* gene. For example, the high expression of TSG-6 in human umbilical cord MSCs attenuates the excessive inflammatory responses induced by severe burns, by inhibiting the activation of P38 and JNK signaling pathways (35).

10 ng/ml TNF-α stimulation was shown to promote TSG-6 release from human bone marrow MSCs (36, 37). Subsequent coculture with macrophages inhibited the NF-κB signaling pathway *via* the expression of CD44, thus, suppressing excessive inflammatory responses (38). Similar studies have also shown that 100 ng/ml TNF-α enhanced the secretion of TSG-6 by human iPSC-MSCs and bone marrow MSCs, initiated TSG6-related inflammatory pathways, mediated hyaluronan and CD44 interactions, suppressed inflammatory responses in an Akt-dependent manner, and promoted epithelial cell proliferation, thereby accelerating mucosal healing in a mouse model of colitis (39).

### The mechanism by which TNF-α regulates the immunosuppressive capacity of MSCs in concert with other factors

An immune response can develop in a pro- or anti-inflammatory direction, depending on the action of various immune cells and inflammatory factors. However, as the first molecule of the inflammatory response, TNF-α assumes a key position in the regulation of multiple immunosuppressive pathways.

#### IL-10 enhances the TNF-α-induced immunosuppressive pathway of MSCs

At present, only one study has reported the cooperation between TNF-α and IL-10 in the immunomodulation of hMSCs. Saldaña et al. showed that human bone marrow MSCs pre-stimulated with 10 ng/ml TNF-α and 1 ng/ml IL-10 were able to significantly reduce TNF-α production by macrophages, compared to MSCs pre-treated with 10 ng/ml TNF-α alone (34). Pre-treating MSCs with 1 ng/ml IL-10 had no effect on TNF-α concentration within the culture medium. This indicates that the role of IL-10 is to promote the TNF-α-induced immunosuppressive capacity of MSCs. Therefore, it would be interesting to investigate: 1) whether pro-inflammatory M1 macrophages secrete TNF-α to stimulate bone marrow MSCs in the early stages of inflammation in the presence of pro-inflammatory factors; and 2) if these MSCs then cause anti-inflammatory M2 macrophages to secrete IL-10 to reduce TNF-α levels by increasing COX-2 and PGE-2 expression. Thus, the inhibition of the inflammatory response is achieved by prompting TNF-α-stimulated MSCs to release PGE-2, which leads to the secretion of IL-10, which in turn synergized with TNF-α. However, how low the concentration of TNF-α needs to drop before this synergistic effect is alleviated, remains unclear.

#### TNF-α enhances the immunosuppressive capacity of IFN-γ-regulated MSCs *via* PD-L1 and IDO

Several published reports have mentioned that TNF-α and IFN-γ act synergistically in the inflammatory microenvironment to increase the expression of various cytokines (e.g., PD-L1, PD-L2, IL-8 (27), IL-6, TGF-β (40), IDO (41), and PGE-2), as well adhesion molecules in hMSCs to enable these cells to perform their immunomodulatory functions. However, the exact synergistic mechanism implicating TNF-α and IFN-γ remains elusive.

It has been reported that TNF-α alone actually reduces the expression of PD-L1 and IDO in murine MSCs. In contrast, IFN-γ, can inhibit T cell proliferation by increasing IDO and PD-L1 expression (42). However, according to a recent study, TNF-α can indirectly promote PD-L1 and IDO expression by increasing IFN-γ levels *via* NF-κB signaling in hMSCs. Thus, TNF-α synergistically amplifies the IFN-γ/STAT signaling pathway (29).

In another study, TNF-α and 10 ng/ml of IL-1β enhanced the expression and sensitivity of the IFN-γ receptor *via* NF-κB signaling in hMSCs (43). Thus, in the presence of TNF-α and IL-1β, IFN-γ could bind to its receptor more easily, activating the signal transducers and activators of transcription (STAT5) and p38-MAPK signaling pathways and leading to the increased release of IL-8 and the recruitment of polymorphonuclear granulocytes (6).

Finally, the pro-inflammatory phenotype of hMSCs induced by pre-stimulation with 0.4 mM sodium palmitate could be reversed by treating the cells with 10 ng/ml IFN-γ and 1 ng/ml TNF-α, causing the MSCs to become anti-inflammatory (44). These anti-inflammatory MSCs were able to inhibit peripheral blood mononuclear cell (PBMC) proliferation through the JAK1/JAK2 signaling pathway.

#### TNF-α and IFN-γ synergistically enhance ICAM-1 expression on the surface of MSCs to strengthen their immunomodulatory capacity

The intercellular adhesion molecule-1 (ICAM-1) can improve the migration ability of 6-8wk old mice MSCs and their adhesion to immune cells (45, 46). In addition, the interaction between ICAM-1 on the surface of human bone marrow MSCs and CD43 on the surface of activated T cells through cell-to-cell contact can inhibit T cell receptor (TCR)-meditated signaling (47), which is one of the most important immunoregulatory mechanisms.

50 ng/ml TNF-α stimulation of human bone marrow MSCs was shown to increase ICAM-1 expression, enhancing MSC migration and their capacity to repair damaged tissues (48). The expression of ICAM-1 also rose when human bone marrow MSCs were stimulated with either 0.5–1 ng/ml TNF-α or 5 ng/ml IFN-γ alone in an inflammatory environment; however, only IFN-γ was able to increase MHC I expression. After co-stimulation of MSCs with 1 ng/ml TNF-α and 5 ng/ml IFN-γ, their MHC I and ICAM-1 expression increased significantly, compared with MSCs subjected to each cytokine alone; however, the mechanism of this synergistic effect remains unclear (25). In brief, TNF-α synergizes with IFN-γ to increase ICAM-1 expression on MSCs, enhancing their immune cell adhesion ability and immunomodulatory capacity.

TNF-α is the first factor released by immune cells in the early stages of inflammation to promote the inflammatory response. This initial stage is followed by a rise in the levels of inflammatory factors such as IFN-γ, which stimulate multiple cells to inhibit the inflammatory process. Although the modes of TNF-α and IFN-γ action *in vitro* are partially understood, the complex inflammatory process *in vivo* is still unclear. Furthermore, investigating the effects of multiple inflammatory factors on the therapeutic efficacy of MSCs during disease progression will be important for future translation into the clinic.

## TNF-α promotes homing of MSCs and enhances tissue repair

Microenvironmental disturbances are the initiating factor for stem cell homing. Various signaling molecules such as chemokines, adhesion molecules, and growth factors are released locally after tissue injury. Before MSCs can perform their functions, they must first migrate to the damaged tissue site (49) and adhere to the microvascular endothelium. On reaching the damaged tissue, MSCs secrete a variety of cytokines such as vascular endothelial growth factor (VEGF) to promote angiogenesis, increase local vascularity (50), and facilitate wound healing.

### TNF-α enhances the migration and adhesion of MSCs

#### TNF-α enhances the migration ability of MSCs

MSCs mobilized from the bone marrow into the peripheral blood migrate to the damaged endothelium and promote endothelial repair. The SDF-1a/CXCR4 signaling pathway plays an important role in the migration of stem cells and tumor cells. Pre-stimulating rats MSCs for 24 h with 50 ng/ml TNF-α was shown to enhance their migration ability by increasing the level of phosphorylated NF-κB-p65 and the expression of CXC chemokine receptor 4 (CXCR4) on the cell surface (51).

Engulfment and cell motility protein 1 (ELMO1) is a core molecule involved in cell migration. ELMO1 binds to dedicator of cytokinesis (DOCK) proteins and participates in the cellular directional migration, neurological development, and cancer cell invasion *via* Rac1. It has been demonstrated that the stimulation of human bone marrow MSCs with high concentrations (100 ng/ml) of TNF-α lowered their expression of METTL14. This led to a reduction in the number of m6A modifications and slowed down the degradation of ELMO1. The binding of ELMO1 to DOCK1 then promoted the activation of Ras-related C3 botulinum toxin substrate1 (Rac1) and thus enhanced the migration capacity of MSCs, as demonstrated in the context of obligate spondylitis disease. Consistent results were obtained both *in vitro* and in *in vivo* experiments in mice (52).

#### TNF-α enhances the adhesion of MSCs to endothelial cells

Vascular endothelial cell adhesion molecule (VCAM-1) is an important cell surface adhesion molecule expressed by a variety of cells, including MSCs (53). It was reported that treating human bone marrow MSCs with 50 ng/ml TNF-α enhanced their adhesion to the vascular endothelium at the site of damage by activating NF-κB, ERK, and JNK signaling pathways and upregulating the expression of VCAM-1 (54, 55). Also, in an ischemic rat injury model, Wistar rats bone marrow MSCs pre-stimulated with 10 ng/ml TNF-α accumulated in muscle tissue in large numbers after tail vein injection (55).

### TNF-α enhances the tissue repair ability of MSCs

MSCs have been shown to promote angiogenesis (56), together with inflammatory response control, which are essential prerequisites for wound healing. Furthermore, MSCs undergo self-renewal and multidirectional differentiation, and can secrete various reparatory factors, including VEGF, fibroblast growth factor (FGF), and matrix metalloproteinase (MMP), which play key roles in the wound healing process.

Co-stimulation of human MSCs with 20 ng/ml TNF-α and 20 ng/ml IL-1β, mimicking an inflammatory environment *in vitro*, transactivated the EGFR-mediated MAPK signaling pathway *via* the TACE/ADAM17 axis. This caused an increase in ERK1/2 phosphorylation and promoted the mRNA and protein expression of FGF2, HGF, HBEGF, and IL-6. Thus, the pre-treatment of MSCs with TNF-α and IL-1β significantly improved wound closure when the MSCs were cocultured with NCI-H292 cells (57).

In 2015, researchers reported that pre-stimulation of mice MSCs with TNF-α could: 1) increase the expression of heme oxygenase-1; 2) enhance paracrine effects; 3) upregulate growth factors (including IGF-1, bFGF, and VEGF), chemokines (including CXCL-3, CCL-2/20, TIMP-2, MMP3, and MMP-2/13), and the immunomodulatory factor TGF-β; and 4) downregulate the inflammatory response. Ultimately, these TNF-α-induced MSCs promoted intestinal stem cell regeneration and increased intestinal epithelial cell proliferation, thus improving the survival of radiation-treated mice (58, 59).

At present, it has been shown that pre-stimulation with TNF-α improves the homing and tissue repair functions of MSCs. However, whether these MSCs are also responsible for tissue repair at the damaged site is still unclear. Thus, the role of TNF-α in MSC homing to the damaged site in an inflammatory environment needs to be further explored.

## The role of TNF-α in MSC differentiation

MSCs have multidirectional differentiation potential. *In vitro* experiments often exploit this feature to differentiate MSCs into osteogenic, lipogenic, and chondrogenic cells, which is the current criterion for the “stemness” of MSCs. However, in inflammatory environments, damaged tissue repair is often accompanied by stem cell differentiation. Thus, the effects of inflammatory factors on MSC differentiation and the underlying mechanisms need to be explored.

### Different concentrations of TNF-α have opposing effects on the osteogenic differentiation of MSCs

Osterix (*OSX*), alkaline phosphatase (*ALP*), and particularly runt-related transcription factor 2 (*RUNX2*) are important osteogenic genes. RUNX2 is an important transcription factor in bone development, playing major regulatory roles in osteoblast differentiation, chondrocyte maturation, and extracellular matrix secretion. Currently, it is believed that TNF-α plays a dual role in the osteogenic differentiation of MSCs. This is because, in the early stages of bone injury, a low concentration of TNF-α promotes osteogenic differentiation and bone tissue repair. Conversely, prolonged exposure to high doses of TNF-α causes bone damage (60).

#### Low concentrations of TNF-α promote the osteogenic differentiation of MSCs

**Figure 3:**
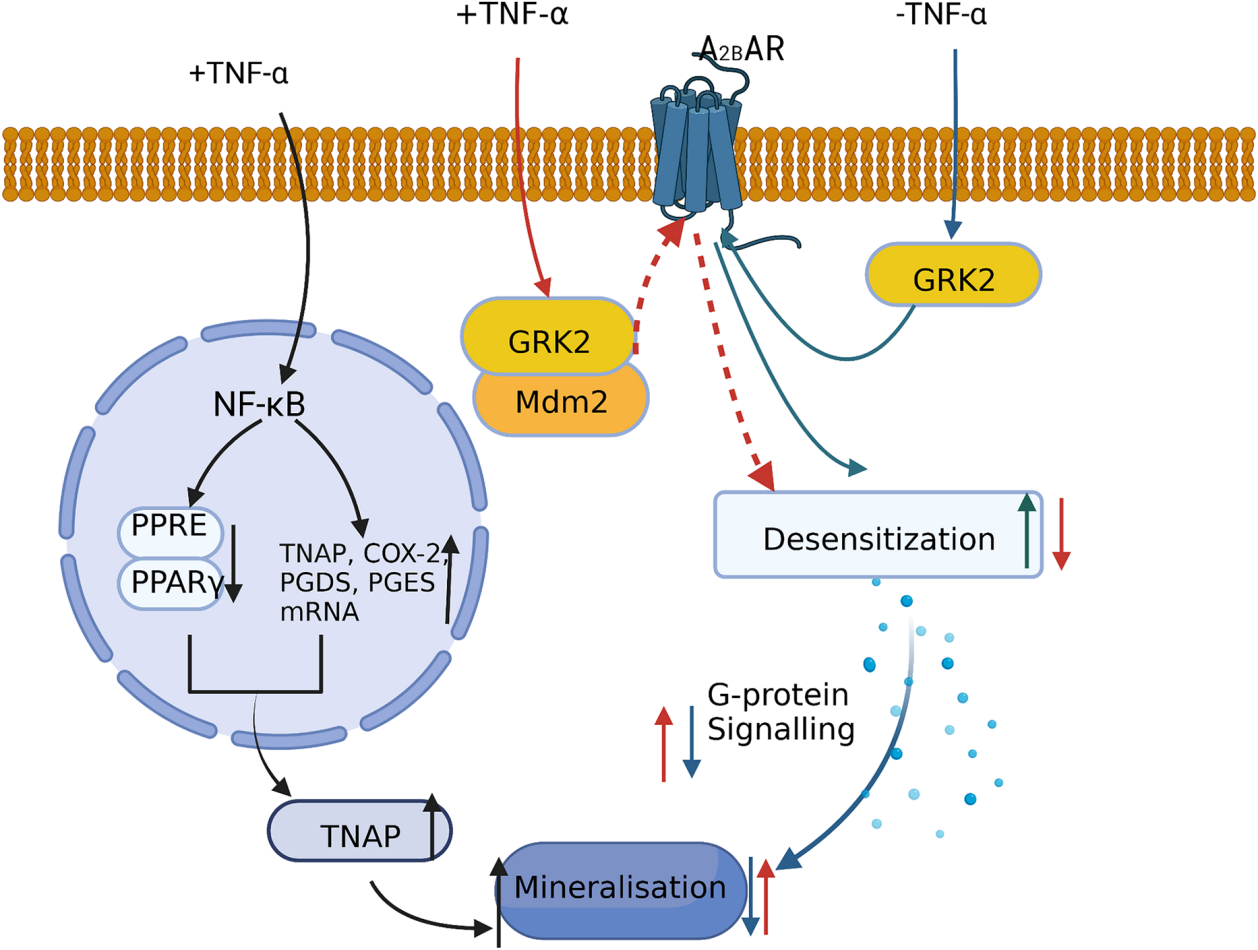
A low concentration of TNF-α promotes MSC mineralization and osteogenic differentiation by: inhibiting PPAR-γ activity and increasing TNAP expression and inducing the ubiquitination and degradation of GRK2 *via* its binding to Mdm2 and decreasing A2BAR desensitization.

Stimulation of human bone marrow MSCs with BAY60-6583, a selective adenosine A2B receptor (A2BAR) agonist, promotes the expression of RUNX2 and ALP and accelerates the osteogenic process (61). However, the binding of G protein-coupled receptor kinase 2 (GRK2) to A2BAR increases the desensitization of A2BAR and reduces MSC mineralization. Treating bone marrow MSCs with a low concentration of TNF-α (1 ng/ml) led to the ubiquitination and degradation of GRK2 by promoting its intracellular binding to murine double minute 2 (Mdm2). This further reduced the BAY60-6583-mediated desensitization of GPCR, leading to an increase in G protein signaling. Finally, both mineralization and calcium deposition were increased, ultimately promoting the osteogenic differentiation of human bone marrow MSCs (60) (**Figure 3**). Moreover, TNF-α and IL-1β can stimulate human bone marrow MSCs in a dose-dependent manner to increase the expression of tissue nonspecific alkaline phosphatase (TNAP), which is required for osteoblast mineralization (62). More specifically, treating human bone marrow MSCs with 1 ng/ml TNF-α was shown to activate the NF-κB pathway, inhibit the binding of peroxisome proliferators-activated receptors (PPAR)-γ and PPRE, and upregulate the mRNA levels of TNAP, COX-2, prostaglandin E2 synthase (PGES), and prostaglandin E2 synthase (PGDS). Thus, *via* this mechanism, TNAP expression was increased in the osteoblast cell line MG-63, which enhanced cell mineralization (63).

#### High concentrations of TNF-α inhibit osteogenic differentiation of MSCs

In the inflammatory environment, TNF-α inhibits osteogenic differentiation through multiple complex signaling pathways. The degree of inhibition may be related to cell type, animal model, duration of action, and immunomodulatory factor concentration. TNF-α can directly or indirectly inhibit the conversion of osteogenic precursor cells to osteoblasts and affect the formation of mineralized nodules (64).

High concentrations of TNF-α were shown to induce the expression of miR-23b and inhibit RUNX2 expression *via* the NF-κB in human bone marrow MSCs (65) or the Wnt/β-catenin signaling pathways in human dental MSCs (66), thus suppressing the osteogenic differentiation of human MSCs. 10 ng/ml TNF-α also inhibited the osteogenic differentiation ability of human periodontal stem cells by decreasing miR-21 expression, leading to a rise in recombinant Sprouty Homolog 1 (SPRY1) levels, thereby suppressing ALP and RUNX2 production (67).

In cranial defect experiments in mice, TNF-α and IL-17 caused the phosphorylation of IκBα and p56, which led to the ubiquitination and degradation of β-catenin in bone marrow MSCs, the inhibition of osteogenic translation factors such as ALP, RUN2, and OSX, and the suppression of osteogenic differentiation of MSCs (68).

**Figure 4:**
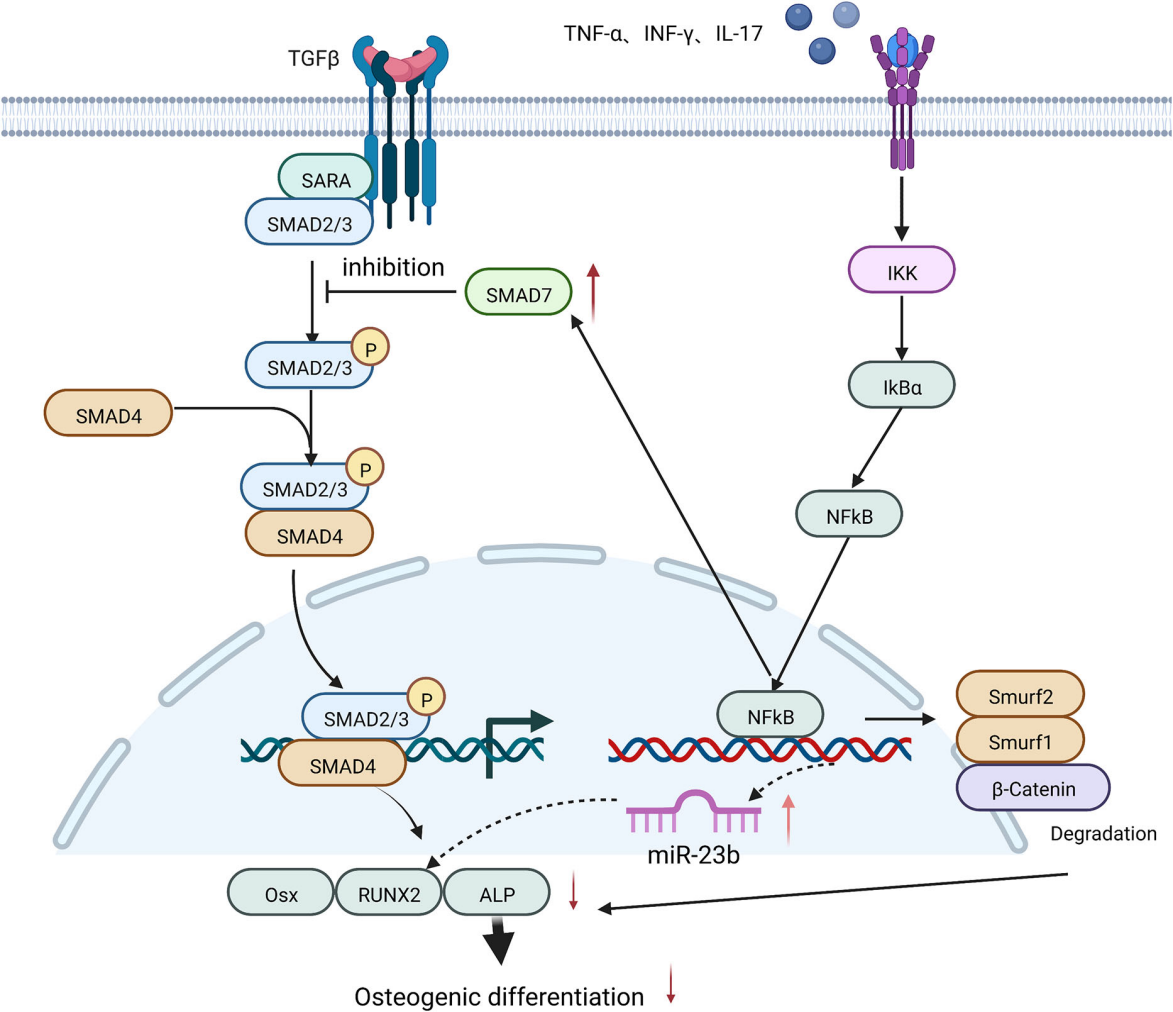
A high concentration of TNF-α inhibits the osteogenic differentiation of MSCs by: increasing the expression of miR-23b and SMAD7; and promoting the degradation of β-Catenin, both of which are mediated by NF-κB signaling and eventually lead to a decrease in the expression of osteogenic-related factors such as RUNX2 and ALP.

In addition, pre-stimulation of bone marrow MSCs from ovariectomized mice (OVE mice) with 5 ng/ml TNF-α and 50 ng/ml IFN-γ activated the NF-κB signaling pathway, increased SMAD7 expression, and lowered RUNX2 and ALP expression. This eventually led to the reduced mineralization of bone marrow MSCs and affected their osteogenic differentiation (19) (**Figure 4**).

Surprisingly, the osteogenic differentiation of hMSCs could also be inhibited by 200 ng/ml IFN-γ through the activation of Fas and the upregulation of Smad6, leading to the downregulation of RUNX2, OCN, and ALP.

### TNF-α inhibits lipogenic differentiation of MSCs

PPAR-γ is a major transcription factor in adipogenesis. 5 ng/ml TNF-α and 5 ng/ml IL-1β, either alone or in combination, can inhibit the lipogenic differentiation of human adipose MSCs by suppressing the expression of PPAR-γ, CAAT-enhancer binding protein α (C/EBP-α), glucose transporters (e.g., GLUT4), and lipoprotein lipase (LPL) (63, 69). Moreover, 10 ng/ml TNF-α could suppress the expression of miR-21 in human periodontal stem cells, increase SPRY1 levels, inhibit PPAR-γ and fatty-acid-binding protein 4 (FABP4) production, and attenuate the lipogenic differentiation of stem cells (67).

### TNF-α inhibits chondrogenic differentiation of MSCs

SRY-Box Transcription Factor 9 (SOX9) is of great significance in the chondrogenic differentiation of MSCs. TNF-α can inhibit the synthesis of SOX9 in human MSCs *via* the NF-κB signaling pathway (70, 71) to inhibit chondrogenesis, while transfection with siRNA targeting TNF-α can restore this differentiation ability of MSCs.

## Antitumor effects of mscs pre-stimulated with TNF-α and associated mechanisms

MSCs are double-edged swords in the process of tumor development (72). On the one hand, MSCs can promote tumor initiation and development by: 1) suppressing the immune response and enhancing the invasiveness of tumor cells (73); 2) secreting multiple trophic factors and cytokines to promote tumor vascularization (74); 3) increasing tumor cell drug resistance by paracrine mechanisms (75); 4) stimulating tumor cells to secrete Bcl-2 and Bcl-XL and inhibiting their apoptosis (76); and 5) transforming into tumor-associated fibroblasts (77). On the other hand, MSCs exhibit anti-tumor capacity by: 1) upregulating the expression of the negative regulators p21 and cyclin D2 to block tumor proliferation and induce apoptosis (78, 79); 2) enhancing the inflammatory response (80); and 3) inhibiting tumor proliferation by inducing Dickkopf-related protein 1 (DKK-1) expression *via* the AKT signaling pathway (81).

However, tumor initiation and progression are often accompanied by a chronic inflammatory response. Under these conditions, as the first molecule of the inflammatory response, TNF-α enhances the anti-tumor capacity of MSCs. Few studies have reported that TNF-α can stimulate MSCs to promote tumor development.

### TNF-α can enhance the inflammatory response and the anti-tumor capacity of MSCs

The Toll-like receptor (TLR) family contains multiple members. TLR2/5/6 activation can induce IL-6 and IL-8 production to initiate the inflammatory response (82), while TLR3 activation can polarize hMSCs towards the anti-inflammatory phenotype *via* the p38 and NF-κB signaling pathways (27).

Stimulation of human adipose MSCs for 96 h with 50 ng/ml TNF-α significantly increased their TLR2 and prostaglandin-endoperoxide synthase 2 (PTGS2) expression, compared to a 24 h stimulation period (59). TLR2 and PTGS2 expression initiated downstream NF-κB signaling pathways, promoting inflammatory responses, and exerting anti-tumor effects. Similarly, the synergy between 1.5 ng/ml TNF-α and 6.5 ng/ml IFN-γ (or 15 ng/ml TNF-α and 65 ng/ml IFN-γ) led to the polarization of bone marrow MSCs towards the pro-inflammatory Th1 phenotype, thus amplifying the anti-tumor immune response. In the tumor inflammatory microenvironment, hMSC polarization can be achieved following the release of TNF-α and INF-γ from activated T cells (25, 83).

### TNF-α enhances the anti-tumor capacity of MSCs by inducing TRAIL expression

It was reported that 24 h coculture of bone marrow MSCs pre-stimulated with 10 ng/ml TNF-α for 48 h with MDA tumor cells, induced tumor cell apoptosis *via* the intercellular interaction with TRAIL on the membrane surface of hMSCs. mRNA or DNA of apoptotic MDA cells in turn promoted the expression of TLR3 in hMSCs, activated the NF-κB signaling pathway, and caused increased TRAIL expression, inducing further apoptosis of TRAIL-sensitive tumor cells (84).

One study also described that the treatment of bone marrow MSCs with 20 ng/ml IFN-γ for 12 h induced the apoptosis of H460 cells *in vitro via* a TRAIL-associated mechanism. However, this was not effectively replicated in animal experiments, as the co-injection of hMSCs and tumor cells greatly enhanced tumor angiogenesis, compared with controls (85).

### TNF-α inhibits the expression of cell proliferation-related factors and enhances the production of tumor suppressor molecules in MSCs after prolonged coculture, suppressing the proliferation of tumor cells

It has been previously reported that TNF-α treatment could further affect the expression of IP10 and RANTES in human bone marrow MSCs after inducing TLR3 expression in these cells (86). IP10 is an important tumor suppressor, which was shown to inhibit tumor angiogenesis by suppressing the formation of artificial blood cell clones, stimulating T cell adhesion to endothelial cells, and enhancing the killing capacity of NK cells (59, 87). Stimulation of human adipose MSCs with 50 ng/ml TNF-α for 96 h (but not 24 h) significantly decreased the expression of factors associated with cell proliferation including VEGF, RANTES, and TGF-β1. In contrast, the levels of IP10 were significantly increased after prolonged TNF-α treatment (88), which inhibited tumor cell proliferation.

### MSCs pre-stimulated with TNF-α induce tumor cell apoptosis by inhibiting checkpoint molecules

**Figure 5:**
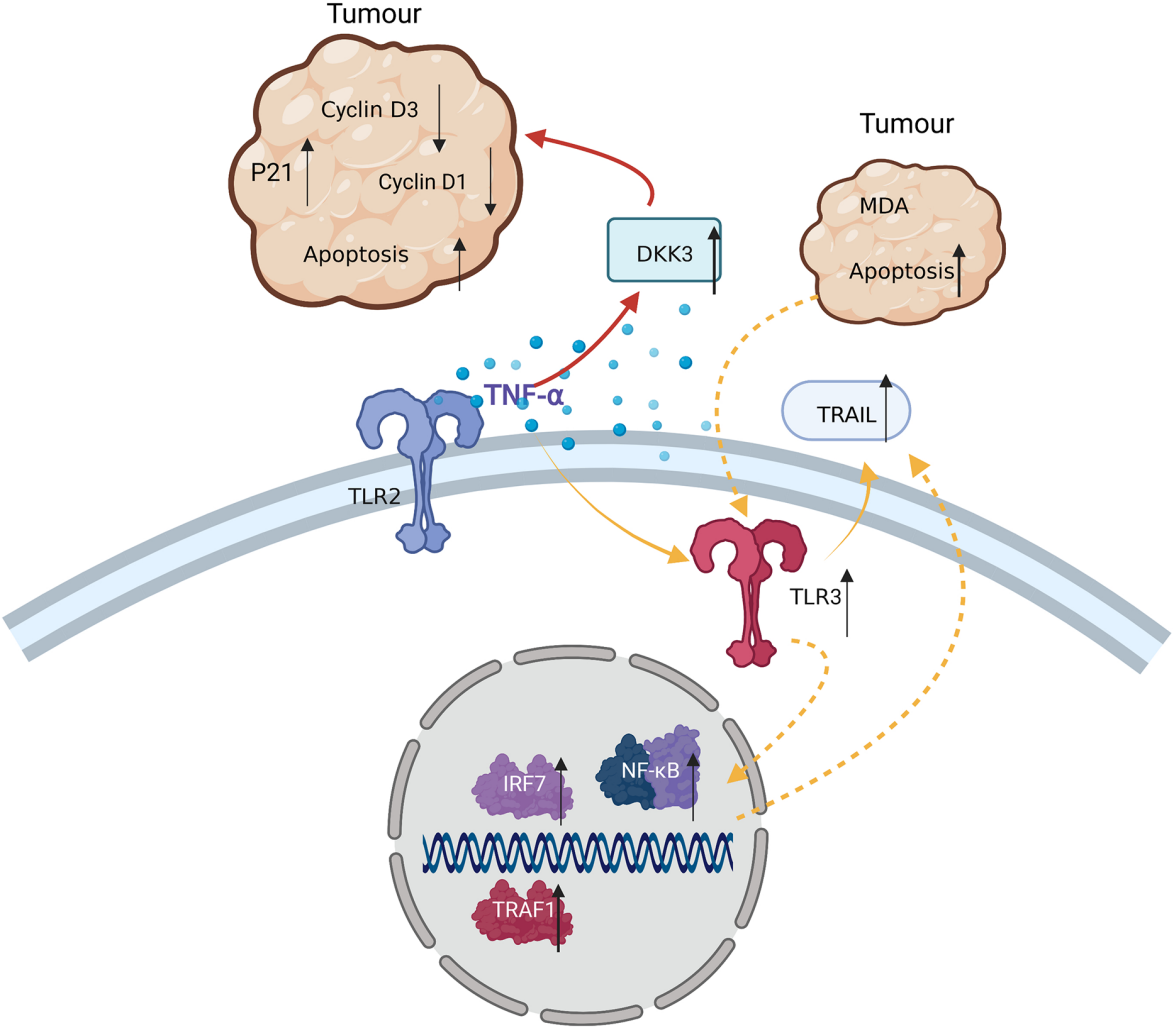
TNF-α exerts anti-tumor effects in TRAIL-sensitive tumor cells by: 1) inducing MDA apoptosis *via* TRAIL and enhancing this process through cascade effects; and 2) inducing MSCs to secrete DKK3, reducing Cyclin D3 and D1 levels in tumor cells, and leading to tumor apoptosis.

Coculture of TNF-α-pre-stimulated human bone marrow MSCs with MDA tumor cells induced tumor cell apoptosis *via* the secretion of DKK3, which inhibited the cyclins D1 and D3, and increased P21 expression in the tumor cells. Gratifying anti-tumor effects were achieved in mice injected with TNF-α-pre-stimulated bone marrow MSCs (84) (**Figure 5**). Therefore, TNF-α-stimulated MSCs have some anti-tumor capacity, which could provide new directions for the treatment of tumors in the future.

However, whether TNF-α stimulation of MSCs in the inflammatory microenvironment or during wound repair induces MSC tumorigenicity, has not been reported. Only one study mentioned that TNF-α can activate p65 to directly activate NF-κB signaling, leading to the release of the pro-inflammatory and pro-cancer factors, CCL-2 and CCL-8, in the inflammatory tumor microenvironment (89). Thus, in this context, TNF-α can recruit a subpopulation of myeloid tumor cells with significant pro-cancer effects.

In summary, TNF-α enhances the anti-tumor capacity of MSCs *via* a variety of mechanisms. Therefore, an in-depth exploration of the role of TNF-α in the tumor microenvironment and its mechanisms will be helpful for the development of future cancer treatment strategies.

## TNF-α affects the composition of MSC exosomal microvesicles

hMSCs can repair damaged tissues by modulating the inflammatory response (90) and reconstituting the microenvironment (91). However, the presence of allogeneic genes in hMSCs (92) and the fact that only 5% of hMSCs reach damaged tissues in the body (93) greatly limits their clinical application. hMSC-derived exosomal microvesicles have MSCs-like immunomodulatory functions and are non-immunogenic, making them promising therapeutic tools (94).

Resting MSCs can autonomously secrete exosomal microvesicles containing cytokines and growth factors including ICAM-1, PGE-2, miR-145, miR-23b, miR-21, miR-146b, miR-133b, and mitochondrial (mt)DNA (95). However, the stimulation of MSCs with multiple factors such as TNF-α and IFN-γ in the inflammatory environment leads to the accumulation of specific components within the exosomal microvesicles. **Table 1** summarizes the changes in the composition or abundance of MSC-derived exosomal microvesicles and how this influences the tissue microenvironment.

**Table 1:** Effect of TNF-α on the exosomal expression profile of MSCs in the inflammatory microenvironment.

Cytokine concentration in inflammatory environment	Stem cell type	Changes in the composition or quantity of exosomal microvesicles	Impact on the tissue microenvironment	References
20 ng/ml TNF-α 20 ng/ml IFN-γ	hBMSCs	Increased: ICAM1, CXCL12, and CCL5 Decreased: IL-5, IL-6, IL-10, and IL-13	Anti-inflammatory	(96)
15 ng/ml TNF-α 10 ng/ml IFN-γ	hBMSCs	Increased: ICAM-1, VCAM-1, and MHC I and II	Enhances the immunomodulatory capacity of MSCs	(97)
15 ng/ml TNF-α 10 ng/ml IFN-γ	hBMSCs	Specific expression of SSEA-4 and CD146	Mediates immune regulation in GVHD *via* Tregs	(98)
25 ng/ml TNF-α 20 ng/ml IL-6 25 ng/ml IL-1β	murine BMSCs	Nearly 3-fold increase in exocrine secretion	Polarizes macrophages to become anti-inflammatory and increases the proportion of Tregs	(99)
10 ng/ml TNF-α	hG-MSCs	Enriched in miR-21-5p	Inhibits PDCD4 expression and protects retinal cells from apoptosis and inflammation	(100)
100 ng/ml TNF-α	hG-MSCs	Increased: IL-1RA and CD73 Specific: miR-1260b	Regulates osteoclastogenesis and reduces periodontal bone loss	(101, 102)
15 ng/ml TNF-α 10 ng/ml IFN-γ	hAd-MSCs	Increased: A20 and TSG-6	Inhibits T cell proliferation *via* dose-dependent effects	(103)
10 ng/ml TNF-α 50 ng/ml IFN-γ 10 ng/ml IL-1β	hDp-MSCs	Increased: IL6, IDO, COX2, and PD-L1	Inhibits T cell proliferation and promotes tissue repair	(104)
20 ng/ml TNF-α 25 ng/ml IFN-γ	hBMSCs	Increased: COX2 and IDO	Polarizes microglia to become anti-inflammatory *in vitro* and inhibits microglial activation *in vivo* (in mice)	(105)
10 ng/ml TNF-α	hUC-MSCs	Enriched in miR-146a	Inhibits the levels of α-SMA, collagen I, collagen III, and mRNA. Suppresses the differentiation of fibroblasts.	(106)
20 ng/ml TNF-α and IFN-γ	hAd-MSCs	Enriched in miR-34a, miR-146a	Promotes polarization of M2 macrophages	(9)

## Conclusion

MSCs have become a major focus of drug transport and cell therapy research because of their low immunogenicity and their ease of isolation, amplification, and differentiation *in vitro (* 107). MSCs have now been used in preclinical and clinical studies to treat a variety of diseases such as diabetes mellitus (108), multiple sclerosis (109), cirrhosis (110), systemic lupus erythematosus (111), Crohn’s disease (112), and GVHD (113). In addition, MSCs have been used to facilitate umbilical cord blood stem cell transplantation (114). Since these MSC-based therapies are influenced by the inflammatory microenvironment, the design of *in vitro* studies or animal models to simulate the *in vivo* inflammatory microenvironment has become a major focus of MSC research (115).

In the inflammatory microenvironment, MSCs experience a gradual decline in function from autophagy to apoptosis. In the early stages of inflammation, low concentrations of TNF-α and other inflammatory factors enable MSCs to survive *via* autophagy. However, as inflammation develops, MSCs are driven towards apoptosis. It has also been reported that autophagy of MSCs in the inflammatory microenvironment could also affect the functions of other cells (116). Thus, the early control of inflammation is important for achieving rapid tissue repair and preserving cell function.

Live, apoptotic, and dead MSCs all have immunomodulatory capacity (117). The effect of TNF-α and other inflammatory factors on MSCs in the tissue microenvironment is a determining factor of their pro- or anti-inflammatory functions. In the non-contact state, TNF-α can both stimulate MSCs to inhibit inflammation (via PGE-2 and TSG-6) and, in the initial stage of inflammation, increase their sensitivity to IFN-γ. This promotes the release of soluble PDL1, PDL2, and IDO from MSCs and inhibits the immune response. In the contact state, PDL1 on the surface of MSCs and PD1 on the surface of T cells act as immune checkpoints (118). TNF-α cooperates with IFN-γ to increase the expression of ICAM-1 and enhance the interaction between MSCs and immune cells, thus allowing PDL1 and CTLA-4 to play a role in immune regulation. Therefore, a comprehensive understanding of the cooperation between various cytokines targeting MSCs in the inflammatory microenvironment provides a basis for the treatment of inflammatory chronic diseases and tumors.

The osteogenic, lipogenic, and chondrogenic differentiation of MSCs is an important manifestation of their “stemness”. Tissue injury is often accompanied by an inflammatory reaction and tissue repair. Under such circumstances, the effect of TNF-α on the differentiation of MSCs will be a non-negligible factor in the clinical application of MSCs. Different TNF-α concentrations, durations of stimulation, and time points of administration may affect the osteogenic differentiation of MSCs. For example, during the development of inflammation, TNF-α inhibits the activity of ALP in the early stages and inhibits the osteogenic differentiation of MSCs by suppressing their mineralization in the later stages (119). In addition, TNF-α can also affect the lipogenic and chondrogenic differentiation of MSCs *via* PPAR-γ and SOX9, respectively; however, the underlying mechanisms are not well understood. Therefore, studying the effects of the inflammatory environment on the differentiation of MSCs is necessary to improve the self-renewal ability of tissues and promote wound healing.

MSCs exert both anti- and pro-tumor effects. However, TNF-α-stimulated MSCs mainly exhibit anti-tumor capacity, principally by: 1) enhancing the inflammatory response to eliminate tumor cells; 2) releasing tumor apoptotic factors, such as IP10 and TRAIL; and 3) blocking the tumor cell cycle. Understanding the synergistic inhibitory effect exerted by TNF-α and MSCs on tumor cells will be useful in designing future anti-tumor cell therapies.

Exosomes or microvesicles play an important role in both the diagnosis and treatment of disease (120), and have the potential to become valuable therapeutic tools in the future (121). In this review, we focused on the differences between the expression profiles of exosomal components within MSCs in the resting and inflammatory states. MSC exosomes or microvesicles have achieved notable success in the treatment of chronic and refractory diseases (122). In addition, TNF-α may modulate the mRNA and protein expression of MSC exosome or microvesicle components for the treatment of a particular disease.

Last, species which MSCs were derived from may be a critical factor affecting the results of animal trials. There were significant differences in the expression of immunosuppressive factors in MSCs from different species. For instance, resting MSCs could produce 60 pg/ml (123) and 90 pg/ml PGE2 (23) when derived from Sprague-Dawley (SD) rats and BALB/c mice, respectively. As for human MSCs, the PGE2 level is 0.2 ng/ml from the human placenta (124), 0.75 ng/ml from the human umbilical cord (125), and 5 ng/ml from the human umbilical cord (purchased from ATCC) (126). Besides PGE2, the level of other cytokines might also be different between MSCs derived from humans and mice, and affect the result of experiments. These results suggested that when we evaluate the efficacy of MSCs, we need to select appropriate evaluation criteria according to the species or tissues of origin, and select the different tissue-derived MSCs according to the pathophysiological characteristics of the disease, which may lead to better efficacy.

The inflammatory microenvironment is complex. TNF-α initiates the inflammatory response and cooperates with multiple factors to participate in inflammation and tissues repair. A greater understanding of the processes and mechanisms underlying the differentiation, regulation, autophagy, and apoptosis of MSCs in the inflammatory microenvironment will provide guidance for future therapeutic strategies involving MSCs.

## Author contributions

XX and JX conceived the article, the first draft of the manuscript was written by WL. JX, XX, critically revised the work. QL, JS, WL, performed the literature search and figure work. All authors contributed to the article and approved the submitted version.
